# Real-time monitoring of military health and readiness: a perspective on future research

**DOI:** 10.3389/fdgth.2025.1542140

**Published:** 2025-05-07

**Authors:** Herman J. de Vries, Sija J. van der Wal, Roos Delahaij, Ward Venrooij, Wim Kamphuis

**Affiliations:** ^1^ Department of Learning and Workforce Development, Netherlands Organisation for Applied Scientific Research (TNO), Soesterberg, Netherlands; ^2^ Department of Human Machine Teaming, Netherlands Organisation for Applied Scientific Research (TNO), Soesterberg, Netherlands

**Keywords:** military personnel, physiological monitoring, wearable technology, biomarkers, psychophysiology, readiness, health status, statistical modeling

## Abstract

Military personnel face significant physical and mental demands, making continuous physiological monitoring essential for understanding health status, managing long-term health risks, and predicting a soldier's readiness to perform in military operations. Recent advancements in wearable technology enable the tracking of biomarkers and psychophysiological indicators, yet current approaches remain fragmented, often focusing on isolated health outcomes rather than comprehensive, actionable insights. This perspective article reviews overarching theoretical health models and examines statistical modeling approaches to better capture the multidimensional nature of health and readiness. Building on these insights, a vision is presented for developing a military health and readiness monitoring system that integrates wearable technology with tailored health indicators and outcomes, aligned with the specific demands of military tasks. The role of advanced tools, such as Large Language Models (LLMs) and Knowledge Graphs in contextualizing health data with operational demands is highlighted, offering a pathway to more accurate and actionable assessments of readiness. This vision outlines key considerations for future development, aiming to empower service members and military leadership with effective tools for health and readiness management.

## 1 Introduction

Military personnel face demanding roles that place significant physical and mental burdens on them (1). While these demands can affect performance in the short term, they may also lead to long-term health issues and attrition (2–5). This concern extends beyond individuals, impacting organizations and society, particularly when armed forces must maintain operational readiness during geopolitical tension in times of personnel shortages (6). Consequently, real-time insight into servicemembers' health and readiness—and understanding factors affecting them—would be very valuable. Such monitoring enables individuals to track their health and readiness, may improve their training or inform medical providers, and can help commanders to make strategic decisions based on current unit-level data on health and readiness (7).

Advancements in wearable sensors have made this vision more attainable. Initially relying on accelerometry, wearables now regularly include photoplethysmography, temperature, and electrodermal activity sensors (8, 9). Epidermal patches monitor biomarkers in sweat, such as lactate, cortisol, glucose and electrolytes (10). Research is expanding from physical activity and sleep tracking to continuous monitoring of mental workload (11), stress (9, 12, 13), resilience (14, 15) and readiness (16) through wearables. This progress enables early detection of infections (17–19) and mental health issues like burnout (20) and depression (21, 22), supporting prevention and improving servicemembers' well-being and readiness (23).

While civilian monitoring technology is rapidly advancing, it is not yet suitable for military real-time health and particularly readiness insight. Within the military context, readiness can be defined as the degree in which the individual is prepared to establish and sustain competent performance in the complex and unpredictable environment of modern military operations (24). Civilian readiness monitoring often focuses on generalized readiness for daily activities or on performance during exercise or sports (25), but in a military context, readiness monitoring needs to be interpretable in relation to mission requirements to be actionable. Researchers have proposed biomarkers for real-time military health and readiness insight (26) and explored artificial intelligence-driven personalized recommendations (27). However, a comprehensive vision is still lacking that outlines which factors to monitor, how these factors interrelate, what the optimal methods for monitoring are, and how data need to be integrated (via models and algorithms) to provide holistic health and readiness assessments in a military context. In this perspective article, we outline how such a vision can be achieved. We begin by reviewing the evolution in theoretical health models, followed by examples of statistical modeling of constructs related to dimensions of health and readiness. Finally, we provide a perspective on what is needed to advance military health and readiness monitoring.

## 2 A brief history of theoretical health and readiness models

In order to determine what aspects of health and readiness are relevant, it is helpful to understand how these concepts are viewed and conceptualized in the literature.

Upon the establishment of the World Health Organization (WHO), health was defined as “*a state of complete physical, mental and social well-being and not merely the absence of disease or infirmity*” (28). This definition departed from the previously dominant biomedical model of health (introduced by Hippocrates in 400 BC), which defined health solely as the absence of disease (29). In 1977, the biopsychosocial model of health was introduced, positing that health is influenced not only by biological factors but also by psychological and social factors (30). This model aligned with the WHO's broader definition and became the dominant perspective in subsequent decades.

During and after the biopsychosocial model's introduction, several other theories emerged that also view health as multi-dimensional. For example, the Health Field Concept divides health into four components: biology, lifestyle, environment, and healthcare organization (31). Similarly, the Socio-Ecological Model emphasizes interactions between individuals and environmental layers, ranging from personal relationships to broader societal factors (32). The Health Determinants Model highlights the influence of individual factors, lifestyle, and contextual conditions such as social networks and socioeconomic factors (33). Hettler's Six Dimensions of Wellness offers a framework for well-being, covering physical, emotional, social, intellectual, occupational and spiritual dimensions (34), later expanded with financial and environmental well-being (35). This framework, often visualized in the Wellness Wheel, is widely used in therapeutic settings. Despite their differing purposes, these models share a focus on contextual and environmental factors complementing the biopsychosocial model.

In 2011, Huber proposed moving away from the WHO's definition, advocating a focus on “*the ability to adapt and self-manage in the face of social, physical, and emotional challenges*” (36), also known as Positive Health. This shifted the focus from an ideal, static state to a dynamic capacity for resilience and adaptation. Huber and colleagues also developed tools for healthcare professionals to implement Positive Health, including a six-dimensional framework encompassing bodily functions, mental well-being, meaningfulness, quality of life, participation and daily functioning (37).

Contrary to the discussed health models, no overarching models for readiness were identified. This can be attributed to the term “readiness” referring to specific aspects, such as psychological readiness (38) or work readiness (39), and to the fact that readiness is a relative concept; it refers to a specific task or mission or assignment. Even definitions of military readiness can be broader than intended for this article, as it can encompass training, equipment and maintenance (40). The importance of these aspects is underlined by the 32-item Acute Readiness Monitoring Scale (ARMS), which assesses current military readiness and includes items on physical, psycho-emotional, cognitive and social readiness to perform (41). Although not all of these aspects of military readiness are broadly covered in academic literature, a theoretical model does exist for operational cognitive readiness (42, 43). This model states that the interplay between knowledge and expertise with cognitive functioning determines operational cognitive readiness, whereas motivation is the driving factor behind all three components.

In summary, the concept of health has evolved from a strictly biomedical approach to a multi-dimensional perspective that includes biological, psychological and social dimensions, along with increasing attention to contextual factors. While some models add dimensions for specific contexts, there is no consensus on a single comprehensive model. The models above suggest dimensions for a holistic, data-driven approach to military health and readiness but do not provide insights into how to model or quantify these concepts. Therefore, the next section focuses on empirical studies, which offer directions for statistical modeling of health and readiness.

## 3 Examples of statistical modeling of health and readiness-related constructs

From the lack of literature on overarching statistical models on health and readiness, it can be concluded that these multidimensional concepts are too complex to model directly. Most studies adopt a narrower focus, modeling outcomes within specific dimensions. These studies provide insights into the factors that need monitoring to create a valid picture of health and readiness subconstructs, which can serve as a basis for a broader model. Each dimension of health and readiness contains many relevant underlying outcomes that can be modelled by an even larger number of predictors. As such, it is outside the scope of this perspective article to provide a complete overview of these outcomes and predictors, but providing some examples of existing research can contribute to the understanding of what is needed to take the next step towards actionable military health and readiness monitoring. As such, the overview below outlines examples of promising studies within several dimensions of health and readiness. Considering the potential for statistical modeling and its relevance to military health and readiness, studies in the physical, psycho-emotional and cognitive dimensions were included. Studies that model outcomes within the social dimension were also considered, but no relevant studies were identified. Military-context studies were first examined, followed by a broader search.

### 3.1 Physical dimension

A number of studies have been published that sought to identify biomarkers that helped explain individual performance readiness. Of special interest is a study in Marines that identified resting heart rate variability (HRV) as a baseline marker for performance readiness, and countermovement jump force production, cognitive psychomotor vigilance and resting HRV as monitoring markers of performance resilience (44). Due to its military relevance, significant progress has also been made in modeling the risk of exertional heat stroke. A recent study showed that a machine learning algorithm using wearable sensor data on heart rate and acceleration can predict exertional heat stroke 33–69 min before collapse with high accuracy (45). Regarding the heavy physical demands on military personnel, relevant models are also found in sports and performance literature. For example, one review proposed sensor data on external and internal training load to optimize performance, assess non-contact injury risk, inform about hydration status to alleviate soft tissue injuries, and assess cardiorespiratory function and capacity (46). Other examples involve predicting physical fatigue using wearable sensors and machine learning, where physical activity (e.g., energy expenditure, steps) and vital signs (e.g., HRV, respiratory rate) are key predictors (47, 48).

### 3.2 Psycho-emotional dimension

Multiple studies have modeled the risk of posttraumatic stress disorder (PTSD) in military personnel. Although wearable sensors are not yet used, these studies offer insights into modeling mental health by combining diverse data sources. One study combined psychological and cognitive functioning, blood values and genetic information to predict probable PTSD after deployment (49). Beyond the military, developments in mental health modeling use sensor data from fitness trackers and mobile phones. For example, studies modeled high vs. low self-reported stress or mental health using fitness tracker data (e.g., heart rate, step count) and mobile phone data (e.g., screen time, timing of calls, text messages) (13, 50, 51). Also, a positive depression screening was linked to data from fitness trackers (e.g., energy expenditure and sleep stages) (52) and self-reported depressed mood was predicted using fitness tracker data (e.g., HRV and sleep duration) in combination with electroencephalography measures (53). Recent advancements in the continuous and objective monitoring of social interactions and support, as utilized in models for psycho-emotional outcomes (54), present opportunities to incorporate the social dimension into data-driven models for health and readiness.

### 3.3 Cognitive dimension

Although few to no examples of cognitive outcome modeling exist in a military context, several studies use wearables and machine learning to predict cognitive fatigue, performance or load in non-military settings (55–57). One study found that physiological data (e.g., blood volume pulse, skin temperature) from wearables correlates with cognitive performance measures like executive function and global cognition (56). Another study showed that sensor data (e.g., heart rate, electrodermal activity) differentiates cognitive load across activities (problem solving, leisure, daydreaming) and levels of self-reported mental focus (55).

### 3.4 Relevance for health and readiness monitoring

The presented overview shows that current health and readiness modeling focuses on specific outcomes within a dimension (e.g., predicting current mood) rather than overarching concepts that could be used to provide more actionable feedback (e.g., psycho-emotional performance capacity). This focus on specific outcomes is unsurprising, as it is easier, yields better results, and is more publishable. Furthermore, current research follows a strict statistical approach with a focus on a limited set of objectively and automatically measured factors. Machine learning algorithms select the combination of factors with the highest predictive value for the subjective construct of interest, thereby ignoring the influence of unmeasured factors. The question remains how useful isolated outcome data is for service members monitoring their health and readiness or military commanders assessing unit readiness.

## 4 Discussion

While recent progress in wearable technology and statistical modeling of specific health- and resilience-related outcomes is promising, the current research landscape remains fragmented and cannot yet provide a sufficient knowledge base for the accurate assessment of health and readiness in a military context. Most existing studies focus on isolated health and readiness outcomes, such as physical fatigue, stress, or cognitive load, without integrating these data into a unified framework. This siloed approach makes it challenging to fully capture the complex interplay of factors influencing the overall health and readiness of military personnel. To address these issues and build on recent advancements, we identify three key gaps and propose an integrated approach to enhance assessments of health and readiness in military contexts.

### 4.1 The need for integrative models

No holistic models of health and readiness that provide a comprehensive overview of relevant factors and describe the relationships between them were identified in our literature search. While theoretical health models have evolved significantly over the years, no single framework explicitly maps the interplay between the underlying factors in ways that are directly relevant for military personnel. Developing such an integrative model would be an essential step in advancing our understanding of health and readiness. By identifying the factors most relevant to military tasks and mapping their interrelations, this model could guide the selection of key wearable indicators or other self-monitoring technologies. It could also offer a framework to interpret statistical model results, situating individual outcomes within a broader perspective of health and readiness. Additionally, this model could help identify critical blind spots, such as physiological, social or environmental factors, that are difficult to monitor but still may influence health and readiness.

### 4.2 Bridging the gap between specific and actionable outcomes

Current research aimed at quantitative modeling of health and readiness focuses on specific outcomes, such as stress or mental well-being, without connecting these to broader constructs of health and readiness that are directly actionable for decision-makers. While these specific outcomes provide valuable insights, they do not fully align with defense organizations' needs for concrete assessments, such as a service member's ability to endure long guard shifts or physically demanding marches. At the same time, modeling every task-specific outcome is infeasible due to the dynamic nature of military operations. To address this, there is a need to define intermediate outcomes that are both general enough to guide decisions across tasks and specific enough for reliable modeling using wearable data and other inputs. For instance, modeling a soldier's cognitive performance capacity would likely be general enough to translate across cognitive activities (e.g., watch duty or decision making), while potentially being specific enough to model based on multimodal data sources. If similar capacity scores can also be modelled for other dimensions such as physical, psycho-emotional and social capacity, the combination of those would together provide a relatively overarching view of the soldier's readiness to perform.

These intermediate outcomes should not only take the individual's capacities into account, but also the specific operation or mission they have to perform. A valid estimation of an individual's readiness can only be achieved by combining the required capacities for a specific task with the individual's existing capacities. Similarly, the potential health consequences of an operation or mission can only be assessed by integrating task-specific information about health threats with person-specific data on health vulnerabilities. Since operational information cannot be directly measured at the individual, additional information about the operation has to be integrated in a different manner in a monitoring system that aims to provide insight in military health and readiness.

### 4.3 Translating data into operational insights

Even with reliable indicators of health and readiness, a critical challenge remains in making these outputs actionable for military decision-makers. Service members and commanders, who operate in high-pressure and rapidly changing environments, require tools that can distill complex data into clear, task-relevant insights. Current monitoring systems often fall short in providing this tailored feedback.

A promising solution involves integrating advanced interpretative tools, such as a hybrid AI approach that combines Large Language Models (LLMs) and Knowledge Graphs (KGs), trained on integrative models and enriched with service members' data. Large Language Models (LLMs) are models that are trained on huge amounts of textual data to understand and generate human-like text. They excel in natural language processing tasks, like summarization and contextual interpretation, making them highly effective at explaining concepts and understanding user questions. KGs, on the other hand, are structured data representations that can store information in a network of interconnected entities and relationships. They enable the retrieval, integration, and connection of datasets.

A hybrid AI approach combines the strengths of LLMs, known for their natural language processing capabilities, and KGs, which excel in retrieving and connecting data. Together, these tools translate statistical outputs, such as calculated scores in health and readiness, into contextualized recommendations. This helps commanders understand how individual or unit-level health and readiness align with specific mission requirements. By offering tailored, adaptive feedback, the combination of LLMs and KGs could significantly enhance the utility of health and readiness monitoring systems for decision-making.

### 4.4 Towards a unified military health and readiness monitoring system

Addressing the identified knowledge gaps can facilitate the development of a soldier health and readiness monitoring system that is grounded in robust fundamental knowledge and also provides practical applicability for defense personnel. By combining theoretical insights, empirical research, and interpretative tools, such a system may help integrate scientific understanding with operational needs.

**Figure 1:**
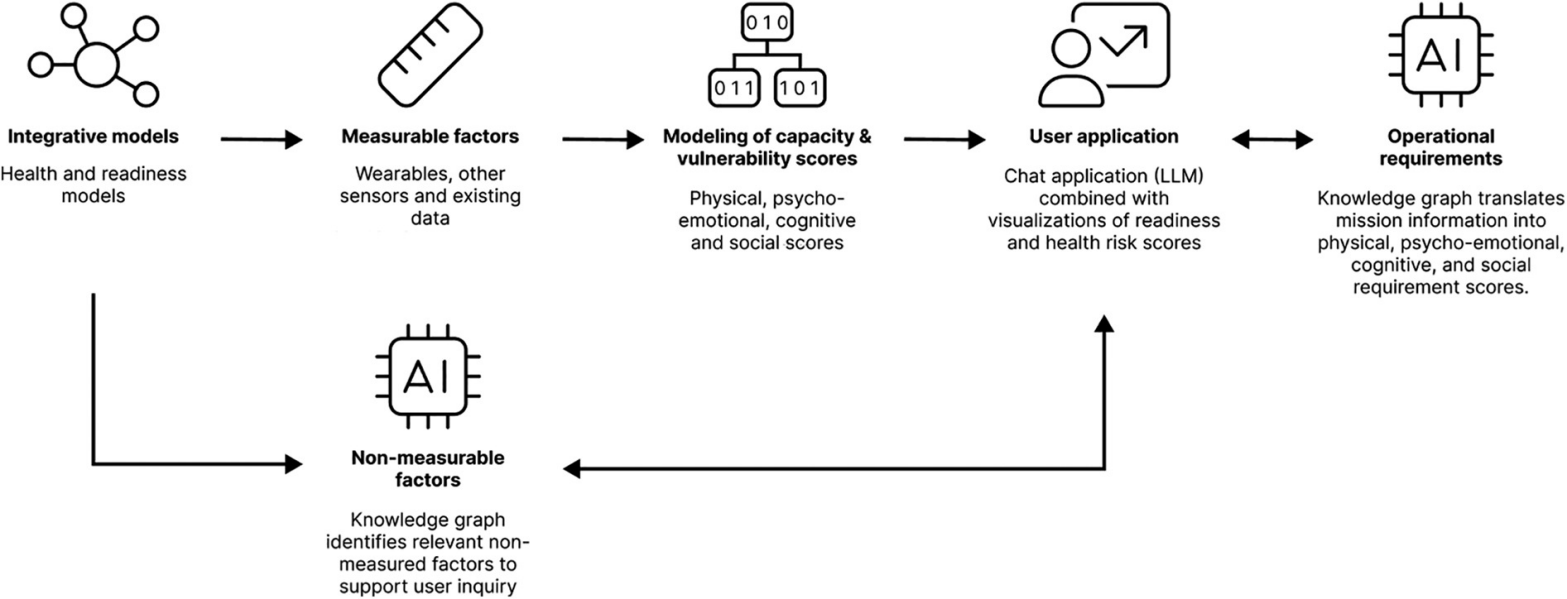
Future soldier health and readiness monitoring system.

Figure 1 displays the structure of our envisioned system. Integrative health and readiness models serve as a foundation for identifying measurable factors at the individual level. These factors could be measured using wearables or other self-monitoring technologies (e.g., brief daily questionnaires on smartphone), or extracted from existing personnel data (e.g., medical records). These factors in turn provide inputs for statistical or machine learning models, which generate scores reflecting the individual's performance capacities and vulnerabilities for health deterioration. These scores are categorized into four dimensions: physical, psycho-emotional, cognitive and social.

To provide the user with relevant and actionable feedback on health risks and readiness for a specific operation or mission, the user application also needs information about the operational requirements (e.g., what health threats is the soldier exposed to, and what performance capacities are required to deal with the operational demands?) and consider factors that are relevant for health and readiness that were not measured (i.e., what aspects of health and readiness might be overlooked by the calculated scores?).

Therefore, to complement the scores resulting from the measurable factors, the system also contains two LLM-based chat applications that can be used to ask the user for additional information. These LLM-based chat applications do not only communicate insights with the user, but can also be used to ask the user about the non-measurable factors and upcoming operational requirements. By using KGs, the system ensures that non-measurable factors stemming from the integrative model are integrated into the user application, and that beside individual capacity and vulnerability scores, the physical, psycho-emotional, cognitive and social *requirement* scores can be calculated for an upcoming mission or operation. Based on a comprehensive analysis of the individual's capacities, vulnerabilities, and the operational demands they face, the (proof of concept) system employs advanced algorithms to synthesize this information into an integrated profile. This profile enhances vulnerability prediction accuracy and supports improved decision-making, enabling users to make informed judgments about a soldier's readiness for military action and associated health risks.

This approach enables users to evaluate whether the performance capacities are sufficient for the coming mission and take potential health risks into account. The dual focus of the system ensures that both short-term operational potential and the trade-offs between immediate deployability and its potential impact on long-term health are considered. By providing tailored insights, the system equips users with a balanced and actionable view of health and readiness, supporting informed decision-making.

## 5 Conclusion

In summary, this work highlights critical gaps in the assessment of health and readiness in military contexts and outlines a pathway to address them. By proposing an approach grounded in integrative models, intermediate outcomes, and interpretative tools, a vision is provided for a system combining theoretical rigor with practical applicability.

Future research should prioritize developing integrative models, identifying actionable intermediate outcomes, and leveraging hybrid AI-tools to personalize and contextualize data. For the future development of these systems, other important aspects to consider for the applicability in military settings are their cost, accessibility, potential reliance on proprietary algorithms and materials, as well as potential privacy, legal and ethical concerns that need to be addressed. These efforts will ensure the envisioned system evolves into a robust, adaptable solution, empowering service members and commanders with actionable insights for sustainable health and readiness management in demanding environments.

## Data Availability

The original contributions presented in the study are included in the article, further inquiries can be directed to the corresponding author.

## References

[1] Boermans SM Kamhuis W Delahaij R Korteling JE(Hans) Euwema MC. Perceived demands during modern military operations. Mil Med. (2013) 178(7):722–28. 10.7205/MILMED-D-12-00463 23820344

[2] Xue C Ge Y Tang B Liu Y Kang P Wang M A meta-analysis of risk factors for combat-related PTSD among military personnel and veterans. PLoS One. (2015) 10(3):e0120270. 10.1371/journal.pone.0120270 25793582 PMC4368749

[3] Pflanz S Sonnek S. Work stress in the military: prevalence, causes, and relationship to emotional health. Mil Med. (2002) 167(11):877–82. 10.1093/milmed/167.11.877 12448610

[4] Buckman JEJ Sundin J Greene T Fear NT Dandeker C Greenberg N The impact of deployment length on the health and well-being of military personnel: a systematic review of the literature. Occup Environ Med. (2011) 68(1):69. 10.1136/oem.2009.054692 20884791

[5] Stevelink SAM Jones M Hull L Pernet D MacCrimmon S Goodwin L Mental health outcomes at the end of the British involvement in the Iraq and Afghanistan conflicts: a cohort study. Br J Psychiatry. (2018) 213(6):690–97. 10.1192/bjp.2018.175 30295216 PMC6429255

[6] Dutch Ministry of Defense. State of Defense—Progress Towards a Future-Proof Armed Forces. The Hague: Dutch Ministry of Defense (2024). https://www.defensie.nl/downloads/publicaties/2024/09/17/stand-van-defensie

[7] Friedl KE. Military applications of soldier physiological monitoring. 4th International Congress on Soldiers’ Physical Performance Vol. 21(11). (2018). p. 1147–53. 10.1016/j.jsams.2018.06.004 29960798

[8] Chandel RS Sharma S Kaur S Singh S Kumar R. Smart watches: a review of evolution in bio-medical sector. 2nd International Conference on Functional Material, Manufacturing and Performances (ICFMMP-2021) Vol. 50(January). (2022). p. 1053–66. 10.1016/j.matpr.2021.07.460

[9] González Ramírez ML García Vázquez JP Rodríguez MD Padilla-López LA Galindo-Aldana GM Cuevas-González D. Wearables for stress management: a scoping review. Healthcare. (2023) 11(17):2369. 10.3390/healthcare11172369 37685403 PMC10486660

[10] Ma J Li H Anwer S Umer W Antwi-Afari MF Xiao EB. Evaluation of sweat-based biomarkers using wearable biosensors for monitoring stress and fatigue: a systematic review. Int J Occup Saf Ergon. (2024) 30:677–703. 10.1080/10803548.2024.2330242 38581242

[11] Wang P Houghton R Majumdar A. Detecting and predicting pilot mental workload using heart rate variability: a systematic review. Sensors. (2024) 24(12):3723. 10.3390/s24123723 38931507 PMC11207491

[12] Bolpagni M Pardini S Dianti M Gabrielli S. Personalized stress detection using biosignals from wearables: a scoping review. Sensors. (2024) 24(10):3221. 10.3390/s24103221 38794074 PMC11126007

[13] Berkemeier L Kamphuis W Brouwer A-M De Vries H Schadd M Van Baardewijk JU Measuring affective state: subject-dependent and -independent prediction based on longitudinal multimodal sensing. IEEE Trans Affect Comput. (2024):1–18. 10.1109/TAFFC.2024.3474098

[14] de Vries H. Wearable and app-based resilience modelling in employees: exploring the possibilities to model psychological resilience using wearable-measured heart rate variability and sleep (Thesis fully internal (DIV)). University of Groningen, Groningen (2023). 10.33612/diss.572706492

[15] Hirten RP Suprun M Danieletto M Zweig M Golden E Pyzik R A machine learning approach to determine resilience utilizing wearable device data: analysis of an observational cohort. JAMIA Open. (2023) 6(2):ooad029. 10.1093/jamiaopen/ooad029 37143859 PMC10152991

[16] de Vries H Oldenhuis H van der Schans C Sanderman R Kamphuis W. Does wearable-measured heart rate variability during sleep predict perceived morning mental and physical fitness? Appl Psychophysiol Biofeedback. (2023) 48:247–57. 10.1007/s10484-022-09578-8 36622531 PMC10195711

[17] Conroy B Silva I Mehraei G Damiano R Gross B Salvati E Real-time infection prediction with wearable physiological monitoring and AI to aid military workforce readiness during COVID-19. Sci Rep. (2022) 12(1):3797. 10.1038/s41598-022-07764-6 35260671 PMC8904796

[18] Hirten RP Tomalin L Danieletto M Golden E Zweig M Kaur S Evaluation of a machine learning approach utilizing wearable data for prediction of SARS-CoV-2 infection in healthcare workers. JAMIA Open. (2022) 5(2):ooac041. 10.1093/jamiaopen/ooac041 35677186 PMC9129173

[19] Pho GN Thigpen N Patel S Tily H. Feasibility of measuring physiological responses to breakthrough infections and COVID-19 vaccine using a wearable ring sensor. Digital Biomarkers. (2023) 7(1):1–6. 10.1159/000528874 37008738 PMC10062187

[20] Barac M Scaletty S Hassett LC Stillwell A Croarkin PE Chauhan M Wearable technologies for detecting burnout and well-being in health care professionals: scoping review. J Med Internet Res. (2024) 26(June):e50253. 10.2196/50253 38916948 PMC11234055

[21] Ahmed A Aziz S Alzubaidi M Schneider J Irshaidat S Serhan HA Wearable devices for anxiety & depression: a scoping review. Computer Methods and Programs in Biomedicine Update. (2023) 3:100095. 10.1016/j.cmpbup.2023.100095 36743720 PMC9884643

[22] Abd-Alrazaq A AlSaad R Shuweihdi F Ahmed A Aziz S Sheikh J. Systematic review and meta-analysis of performance of wearable artificial intelligence in detecting and predicting depression. Npj Digital Medicine. (2023) 6(1):84. 10.1038/s41746-023-00828-5 37147384 PMC10163239

[23] Winslow BD Kwasinski R Hullfish J Ruble M Lynch A Rogers T Automated stress detection using mobile application and wearable sensors improves symptoms of mental health disorders in military personnel. Frontiers in Digital Health. (2022) 4:919626. 10.3389/fdgth.2022.919626 36082233 PMC9445306

[24] Fletcher JD Wind AP. The evolving definition of cognitive readiness for military operations. In: O’Neil HF Perez RS Baker EL, editors. Teaching and Measuring Cognitive Readiness. Boston, MA: Springer US (2014). p. 25–52. 10.1007/978-1-4614-7579-8_2

[25] Ibrahim AH Beaumont CT Strohacker K. Exploring regular exercisers’ experiences with readiness/recovery scores produced by wearable devices: a descriptive qualitative study. Appl Psychophysiol Biofeedback. (2024) 49(3):395–405. 10.1007/s10484-024-09645-2 38668986

[26] Koltun KJ Bird MB Forse JN Nindl BC. Physiological biomarker monitoring during arduous military training: maintaining readiness and performance. Journal of Science and Medicine in Sport. (2022) 26:S64–70. 10.1016/j.jsams.2022.12.005 36631385

[27] Winslow BD Mills E. Future of service member monitoring: the intersection of biology, wearables and artificial intelligence. BMJ Military Health. (2023) 170:412–4. 10.1136/military-2022-002306 36702525

[28] World Health Organization. Constitution of the World Health Organization (1948). Available at: https://www.who.int/about/governance/constitution (Accessed November 12, 2024).

[29] Willis KF Elmer SL. Society, Culture and Health-an Introduction to Sociology for Nurses. Melbourne: Oxford University Press (2007).

[30] Engel GL. The need for a new medical model: a challenge for biomedicine. Science. (1977) 196(4286):129–36. 10.1126/science.847460 847460

[31] Tulchinsky TH. Chapter 21—Marc lalonde, the health field concept and health promotion. In: Tulchinsky TH, editor. Case Studies in Public Health. London: Academic Press (2018). p. 523–41. 10.1016/B978-0-12-804571-8.00028-7

[32] Bronfenbrenner U. The Ecology of Human Development: Experiments by Nature and Design. London: Harvard University Press (1979).

[33] Whitehead M Dahlgren G. What can be done about inequalities in health? Originally Published as. (1991) 2(8774):1059–63. 10.1016/0140-6736(91)91911-D 1681366

[34] Hettler B Weston C Carini J Amundson J. Wellness promotion on a university campus. Family and Community Health. (1980) 3(1):77–95. 10.1097/00003727-198005000-00008 10246133

[35] Stoewen DL. Dimensions of wellness: change your habits, change your life. The Canadian Veterinary Journal=La Revue Veterinaire Canadienne. (2017) 58(8):861–62. 28761196 PMC5508938

[36] Huber M André Knottnerus J Green L van der Horst H Jadad AR Kromhout D How should we define health? Br Med J. (2011) 343(July):d4163. 10.1136/bmj.d4163 21791490

[37] Institute for Positive Health. Positieve Gezondheid—Stichting Institute for Positive Health (iPH). Utrecht: Stichting Institute for Positive Health (2024). https://www.iph.nl/en/

[38] Boitzova A Simonova N. “Psychological readiness”. Definition and approaches. In: Proceedings of the II International Scientific-Practical Conference “Psychology of Extreme Professions” (ISPCPEP 2019). Dordrecht: Atlantis Press (2019). p. 21–4. 10.2991/ispcpep-19.2019.5

[39] Peersia K Rappa NA Perry LB. Work readiness: definitions and conceptualisations. High Educ Res Dev. (2024) 43(8):1830–45. 10.1080/07294360.2024.2366322

[40] Harrison T. Rethinking readiness. SSQ. (2014) 8(3):38–68. Available at: http://www.jstor.org/stable/26270619

[41] Keegan RJ Flood A Niyonsenga T Welvaert M Rattray B Sarkar M Development and initial validation of an acute readiness monitoring scale in military personnel. Front Psychol. (2021) 12:738609. 10.3389/fpsyg.2021.738609 34867619 PMC8636321

[42] Crameri L Hettiarachchi I Hanoun S. A review of individual operational cognitive readiness: theory development and future directions. Hum Factors. (2021) 63(1):66–87. 10.1177/0018720819868409 31424956

[43] Grier RA. Military cognitive readiness at the operational and strategic levels: a theoretical model for measurement development. J Cogn Eng Decis Mak. (2012) 6(4):358–92. 10.1177/1555343412444606

[44] Thompson AG Ramadan JH Alexander JS Galster SM. Psychophysiology, cognitive function, and musculoskeletal status holistically explain tactical performance readiness and resilience. The Journal of Strength & Conditioning Research. (2023) 37(12):2443–56. 10.1519/JSC.0000000000004580 38015734

[45] Yaldiz CO Buller MJ Richardson KL An S Lin DJ Satish A Early prediction of impending exertional heat stroke with wearable multimodal sensing and anomaly detection. IEEE J Biomed Health Inform. (2023) 27(12):5803–14. 10.1109/JBHI.2023.3323014 37812534

[46] Seshadri DR Thom ML Harlow ER Gabbett TJ Geletka BJ Hsu JJ Wearable technology and analytics as a complementary toolkit to optimize workload and to reduce injury burden. Front Sports Act Living. (2021) 2:630576. 10.3389/fspor.2020.630576 33554111 PMC7859639

[47] Sedighi Maman Z Ali Alamdar Yazdi M Cavuoto LA Megahed FM. A data-driven approach to modeling physical fatigue in the workplace using wearable sensors. Appl Ergon. (2017) 65(November):515–29. 10.1016/j.apergo.2017.02.001 28259238

[48] Luo H Lee P-A Clay I Jaggi M De Luca V. Assessment of fatigue using wearable sensors: a pilot study. Digital Biomarkers. (2020) 4(Suppl 1):59–72. 10.1159/000512166 33442581 PMC7768149

[49] Schultebraucks K Qian M Abu-Amara D Dean K Laska E Siegel C Pre-deployment risk factors for PTSD in active-duty personnel deployed to Afghanistan: a machine-learning approach for analyzing multivariate predictors. Mol Psychiatry. (2021) 26(9):5011–22. 10.1038/s41380-020-0789-2 32488126 PMC8589682

[50] Magal N Rab SL Goldstein P Simon L Jiryis T Admon R. Predicting chronic stress among healthy females using daily-life physiological and lifestyle features from wearable sensors. Chronic Stress. (2022) 6(January):24705470221100987. 10.1177/24705470221100987 35911618 PMC9329827

[51] Sano A Taylor S McHill AW Phillips AJ Barger LK Klerman E Identifying objective physiological markers and modifiable behaviors for self-reported stress and mental health status using wearable sensors and mobile phones: observational study. J Med Internet Res. (2018) 20(6):e210. 10.2196/jmir.9410 29884610 PMC6015266

[52] Rykov Y Thach T-Q Bojic I Christopoulos G Car J. Digital biomarkers for depression screening with wearable devices: cross-sectional study with machine learning modeling. JMIR Mhealth Uhealth. (2021) 9(10):e24872. 10.2196/24872 34694233 PMC8576601

[53] Shah RV Grennan G Zafar-Khan M Alim F Dey S Ramanathan D Personalized machine learning of depressed mood using wearables. Transl Psychiatry. (2021) 11(1):338. 10.1038/s41398-021-01445-0 34103481 PMC8187630

[54] Bolliger L Lukan J Luštrek M De Bacquer D Clays E. Protocol of the STRess at work (STRAW) project: how to disentangle day-to-day occupational stress among academics based on EMA, physiological data, and smartphone sensor and usage data. Int J Environ Res Public Health. (2020) 17(23):8835. 10.3390/ijerph17238835 33561061 PMC7730921

[55] Romine WL Schroeder NL Graft J Yang F Sadeghi R Zabihimayvan M Using machine learning to train a wearable device for measuring students’ cognitive load during problem-solving activities based on electrodermal activity, body temperature, and heart rate: development of a cognitive load tracker for both personal and classroom use. Sensors. (2020) 20(17):4833. 10.3390/s20174833 32867055 PMC7506959

[56] Rykov Y Patterson M Gangwar B Jabar S Leonardo J Ng KP Predicting cognitive scores from wearable-based digital physiological features using machine learning: data from a clinical trial in mild cognitive impairment. BMC Med. (2024) 22(1):36. 10.1186/s12916-024-03252-y 38273340 PMC10809621

[57] Varandas R Lima R Badia SBI Silva H Gamboa H. Automatic cognitive fatigue detection using wearable fNIRS and machine learning. Sensors. (2022) 22(11):4010. 10.3390/s22114010 35684626 PMC9183003

